# Cover crop residue decomposition triggered soil oxygen depletion and promoted nitrous oxide emissions

**DOI:** 10.1038/s41598-024-58942-7

**Published:** 2024-04-10

**Authors:** Facundo Lussich, Jashanjeet Kaur Dhaliwal, Anthony M. Faiia, Sindhu Jagadamma, Sean M. Schaeffer, Debasish Saha

**Affiliations:** 1 Department of Biosystems Engineering and Soil Science, University of Tennessee, Knoxville, TN 37996 USA; 2 Department of Earth and Planetary Sciences, University of Tennessee, Knoxville, TN 37996 USA

**Keywords:** Biogeochemistry, Environmental sciences

## Abstract

Cover cropping is a promising strategy to improve soil health, but it may also trigger greenhouse gas emissions, especially nitrous oxide (N_2_O). Beyond nitrogen (N) availability, cover crop residue decomposition may accelerate heterotrophic respiration to limit soil O_2_ availability, hence promote N_2_O emissions from denitrification under sub-optimal water-filled pore space (WFPS) conditions that are typically not conducive to large N_2_O production. We conducted a 21-day incubation experiment to examine the effects of contrasting cover crop residue (grass vs legume) decomposition on soil O_2_ and biogeochemical changes to influence N_2_O and CO_2_ emissions from ^15^N labeled fertilized soils under 50% and 80% WFPS levels. Irrespective of cover crop type, mixing cover crop residue with N fertilizer resulted in high cumulative N_2_O emissions under both WFPS conditions. In the absence of cover crop residues, the N fertilizer effect of N_2_O was only realized under 80% WFPS, whereas it was comparable to the control under 50% WFPS. The N_2_O peaks under 50% WFPS coincided with soil O_2_ depletion and concomitant high CO_2_ emissions when cover crop residues were mixed with N fertilizer. While N fertilizer largely contributed to the total N_2_O emissions from the cover crop treatments, soil organic matter and/or cover crop residue derived N_2_O had a greater contribution under 50% than 80% WFPS. Our results underscore the importance of N_2_O emissions from cover crop-based fertilized systems under relatively lower WFPS via a mechanism of respiration-induced anoxia and highlight potential risks of underestimating N_2_O emissions under sole reliance on WFPS.

## Introduction

Global agriculture annually generates approximately 3.8 billion Mg of crop residues, of which 4.8 million Mg is contributed by the U.S.^1^. Increasing adoption of winter cover crops as a soil health practice will continue generating organic residues. For example, U.S. cover crop acres in 2017 (15.4 million acres) was 50% higher than that in 2012 (10.3 million acres)^2^. When cover and cash crop residues are returned back to the soils, they can provide diverse ecosystem services including soil carbon (C) sequestration^3,4^ and overall improvement of soil health^5–8^. However, decomposing fresh residues can influence coupled soil C and nitrogen (N) cycling, which could be important with regards to the emission of nitrous oxide (N_2_O)^9–13^; a long-lived potent greenhouse gas (GHG) largely emitted from global agricultural soils^14^. This process has the potential to be amplified when winter cover crops are used, as their decomposition upon termination often coincides with N fertilization before summer crop planting in many agricultural production systems.

Cover crop residue quality determines N release during decomposition, with high-quality residues (low C:N ratio, e.g., legumes) often exhibit faster mineralization and N release than high C:N ratio non-legume residues^15,16^. The cover crop residue influence on N_2_O emissions is far from straightforward and further depends on management practices (termination method, N fertilization source and rate) and environmental conditions^9,17^. Beyond N supply, simultaneous increase in C availability during residue decomposition can trigger heterotrophic microbial respiration, leading to rapid soil oxygen (O_2_) consumption. Under such conditions, water induced O_2_ diffusion limitation may not be required to prevail anoxic conditions and N_2_O emissions^18^. While N_2_O emissions in response to residue addition have been widely attributed to altered C and N availability^19^, wetness independent anoxia during residue decomposition as a possible mechanism of N_2_O production has only been postulated with limited direct evidence^11,20^. The challenge lies in the lack of an effective approach to capture high-resolution soil O_2_ consumption in the pore spaces where critical C and N cycling processes occur to trigger N_2_O emissions.

Although the relevance of nitrification and denitrification processes as pathways of soil N_2_O production is well-established^21^, multiple microbial pathways of N_2_O production can often co-occur in the soil^22^. Typically, microbial denitrification is one of the dominant pathways of soil N_2_O production, in which N_2_O is produced as an intermediate product during stepwise reduction of nitrate (${\text{NO}}_{3}^{ - }$) to di-nitrogen (N_2_) gas under anoxic conditions^23^. Complex interactions between O_2_ concentration in soil microsites and ${\text{NO}}_{3}^{ - }$ and C availability regulate not only the total amount of denitrification but also the ratio between N_2_ and N_2_O produced^24–26^. Under limited soil O_2_ but abundant ${\text{NO}}_{3}^{ - }$ supply, increasing C availability from residue decompositions could stimulate heterotrophic denitrification with N_2_O being the dominant product^27^. Therefore, when cover crop residues and N fertilizer co-occur in space and time, decomposing cover crop residues could enhance N_2_O losses from N fertilizer by providing C for energy to microbes^28^ and increased heterogeneity in soil O_2_ availability around the decomposing residues, a key control for denitrification^18,29^.

While spatial distribution of soil O_2_ has long been recognized as a proximal driver of N_2_O production^30,31^, its high-resolution measurements are difficult under field conditions. As a surrogate for O_2_ availability, water-filled pore space (WFPS) is often used to link N_2_O production with soil O_2_ availability^32–34^. It is generally accepted that soil N_2_O emissions from nitrification occur within the range of 30–60% WFPS content, whereas denitrification dominates at higher WFPS (60–100%), with N_2_O as the major product up to 80% WFPS and N_2_ with increasing WFPS thereafter^34,35^. Despite this prevailing consensus, certain studies have identified a decoupling of the relationship between WFPS and N_2_O production pathways, where changes in WFPS did not have the anticipated impact on N_2_O source partitioning^36,37^. Soil WFPS only accounts for diffusion limitation due to soil wetness and neglects soil O_2_ depletion due to accelerated heterotrophic respiration that could create anoxia to promote denitrification even under lower WFPS conditions, otherwise not conducive for denitrification and significant N_2_O emissions^38,39^. Given abundant ${\text{NO}}_{3}^{ - }$ supply, those conditions could trigger equally high N_2_O emissions as in diffusion induced O_2_ limitation under high WFPS that could quickly escape to the atmosphere due to greater diffusivity under lower WFPS. Further understanding of the interplay among N fertilizer management, cover cropping, and N_2_O emissions in cropping systems is imperative due to the increasing adoption of soil health practices such as cover cropping, which promotes residue addition to soils, alongside the concerning trend in increasing atmospheric N_2_O concentration in the recent decades^40,41^. This is crucial to effectively assess net GHG mitigation potential of cover crops as elevated N_2_O emissions can offset soil C sequestration benefits^42,43^, leading agricultural systems to shift from net C sink to a C source.

In a laboratory microcosm study, we investigated the effects of contrasting cover crop residue (grass vs legume) decomposition on high-resolution soil O_2_ dynamics to influence N_2_O emissions from N fertilized soils under different WFPS levels (50% and 80%). We hypothesized that accelerated O_2_ depletion caused by heterotrophic respiration in the presence of decomposing cover crop residues will create O_2_ limited conditions that promote N_2_O emissions even under WFPS conditions that are sub-optimal for denitrification and high N_2_O emissions. We also hypothesized that in presence of N fertilizer, high-quality vetch cover crop residue will deplete soil O_2_ at a much faster rate than wheat cover crop residue and produce greater N_2_O emissions from N fertilizer.

## Results

### Cover crop residue and N fertilization impacts on temporal N_2_O and CO_2_ emissions

**Figure 1:**
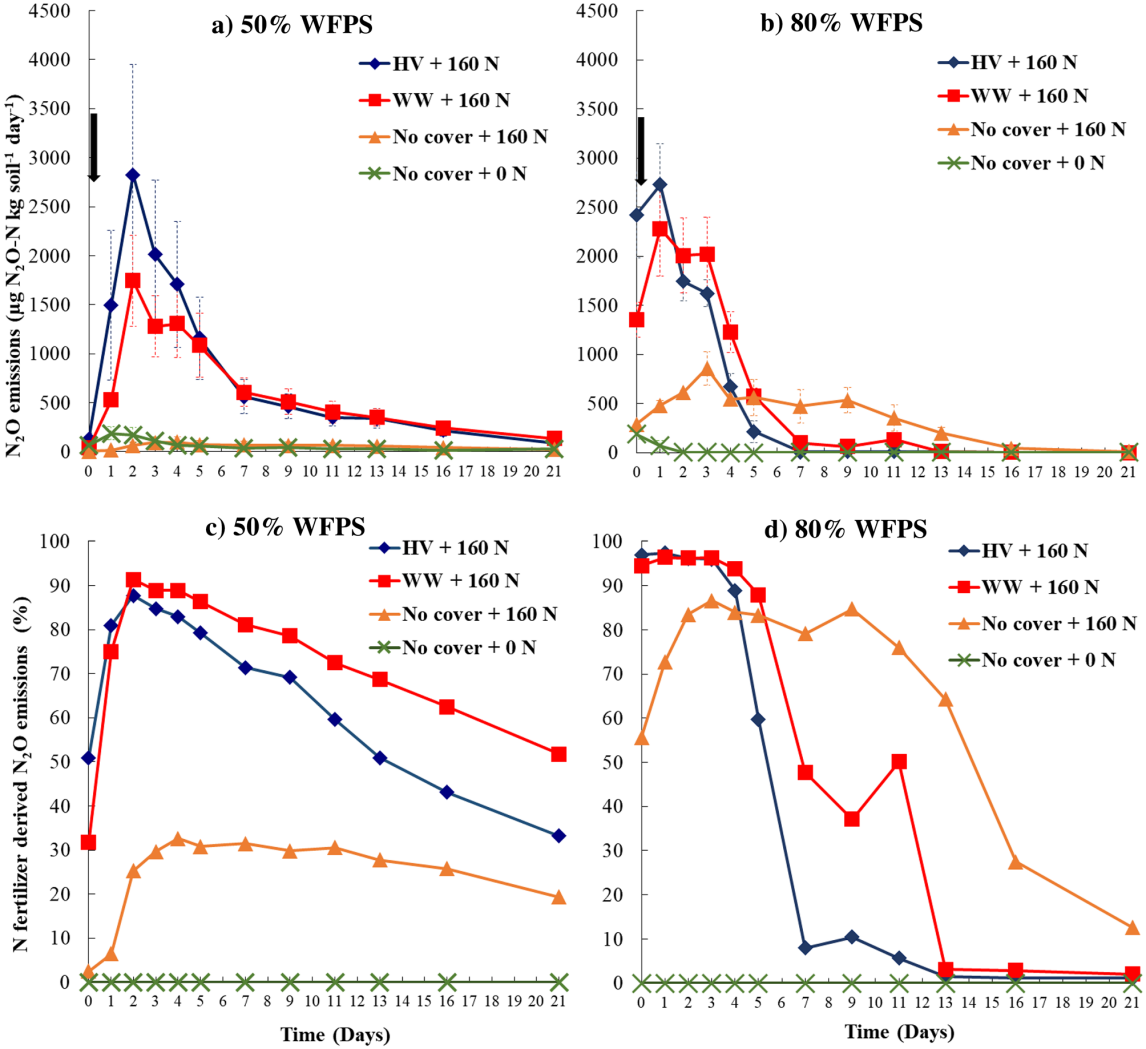
Daily nitrous oxide (N_2_O) emissions (**a**, **b**) and N_2_O emissions derived from N fertilizer (**c**, **d**) over the incubation period from four cover crops treatments at 50% WFPS (**a**, **c**), and 80% WFPS (**b**, **d**). Nitrogen fertilizer was added at the beginning of the incubation experiment as indicated by black arrow in panels (**a**) and (**b**). Bars in panels (**a**) and (**b**) indicate mean standard error. HV, Hairy vetch; WW, Winter wheat; No cover, No cover crop; No cover + 0 N, Control.

The combination of cover crop residue and N fertilizer addition exhibited higher peak N_2_O emissions than the N fertilized without cover crop and control treatments under both 50% and 80% WFPS conditions (Fig. 1a,b). Temporal N_2_O emissions showed different trends across cover crop treatments under 50% and 80% WFPS. First, cover crop treatments under 80% WFPS immediately showed high daily emissions ranging from 674 to 2728 µg N_2_O–N kg soil^−1^ day^−1^ during day 0–4. Whereas peak N_2_O emissions were slightly delayed under 50% WFPS ranging from 529 to 2824 µg N_2_O–N kg soil^−1^ day^−1^, the same or slightly higher in magnitude than that under 80% WFPS. Second, N_2_O emissions from the cover crop treatments sharply decreased following the peak emissions under 80% WFPS, with identical emissions to the control treatment after day 7. In contrast, the decline in emissions were more gradual under 50% WFPS where moderate daily N_2_O emissions (ranging from 88 to 607 µg N_2_O-N kg soil^−1^ day^−1^), significantly higher than the control treatment (*p* < 0.05), were observed until the end of the incubation experiment. Third, in the absence of cover crop residues, the N fertilized treatment produced very little N_2_O emissions under 50% WFPS, about the same as the control treatment. In contrast, N fertilization under 80% WFPS had significantly higher N_2_O emissions than the control treatment from day 1 to day 16; however, the peak emissions were much lower in magnitude (ranging from 44 to 859 µg N_2_O–N kg soil^−1^ day^−1^) and showed a slightly different emission patterns than the cover crop treatments. Lastly, residue type (legume vs grass) effect on temporal N_2_O emissions was not consistent across the WFPS treatments, with higher peak emissions from hairy vetch than winter wheat residue addition with N fertilizer under 50% WFPS (*p* > 0.05).

The temporal variability of N_2_O emissions derived from N fertilizer showed different patterns across WFPS and cover crop treatments that were consistent with the temporal N_2_O emission trends (Fig. 1c, d). Firstly, in the cover crops treatments under 80% WFPS, a high proportion of N_2_O emissions was derived from N fertilizer, ranging from 89 to 97% during day 0 to 4, coinciding with peak N_2_O emissions. The contribution sharply decreased after day 5, concomitant with the decline in total N_2_O emissions. Similarly, in the same treatments under 50% WFPS, N_2_O emissions derived from N fertilizer closely followed the daily N_2_O emission pattern, accounting for 79 to 91% of total N_2_O emissions during day 2 to 5, and gradually decreased to 33% by the end of the incubation experiment. Secondly, in the absence of cover crop residues under 50% WFPS, around one-third of the total N_2_O emissions was derived from N fertilizer after day 2, and it remained relatively constant until the end of the experiment. Nitrogen fertilizer derived N_2_O under 80% WFPS from only N fertilized treatment comprised a much greater proportion (50–80%) to the total emission from day 1 to 16.

**Figure 2:**
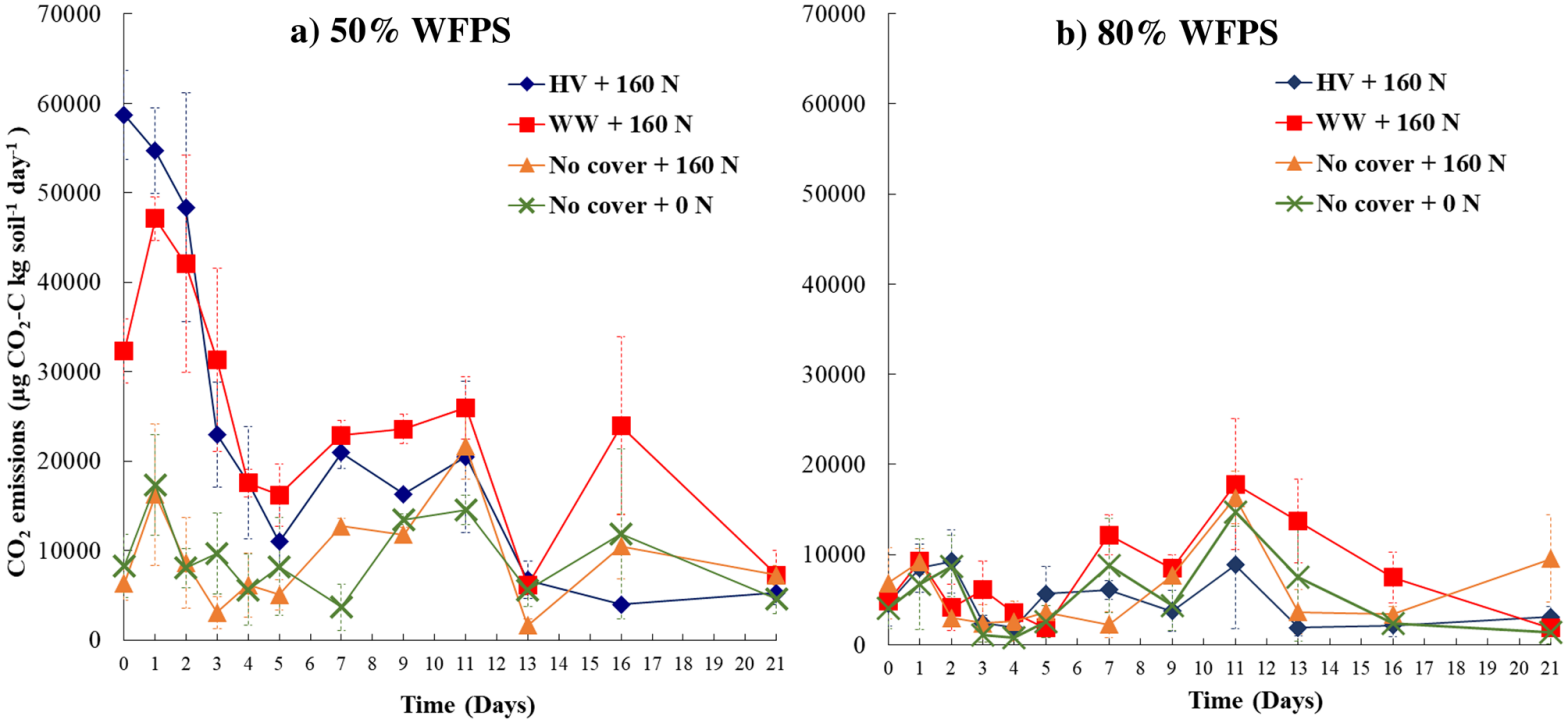
Daily carbon dioxide (CO_2_) emissions over the incubation period from four cover crops treatments at (**a**) 50% WFPS, and (**b**) 80% WFPS. Bars represents mean standard error. HV, Hairy vetch; WW, Winter wheat; No cover, No cover crop; No cover + 0 N, Control.

Soil CO_2_ emissions were higher under cover crop treatments at 50% WFPS (Fig. 2), especially until day 4 (ranging from 17,541 to 58,734 µg CO_2_–C kg soil^−1^ day^−1^), than the non-cover crop treatments (N fertilizer and control). Treatments under 80% WFPS exhibited lower CO_2_ emissions (764–16,329 µg CO_2_–C kg soil^−1^ day^−1^) than under 50% WFPS and did not differ among the treatments.

### Cumulative N_2_O and CO_2_ emissions

**Figure 3:**
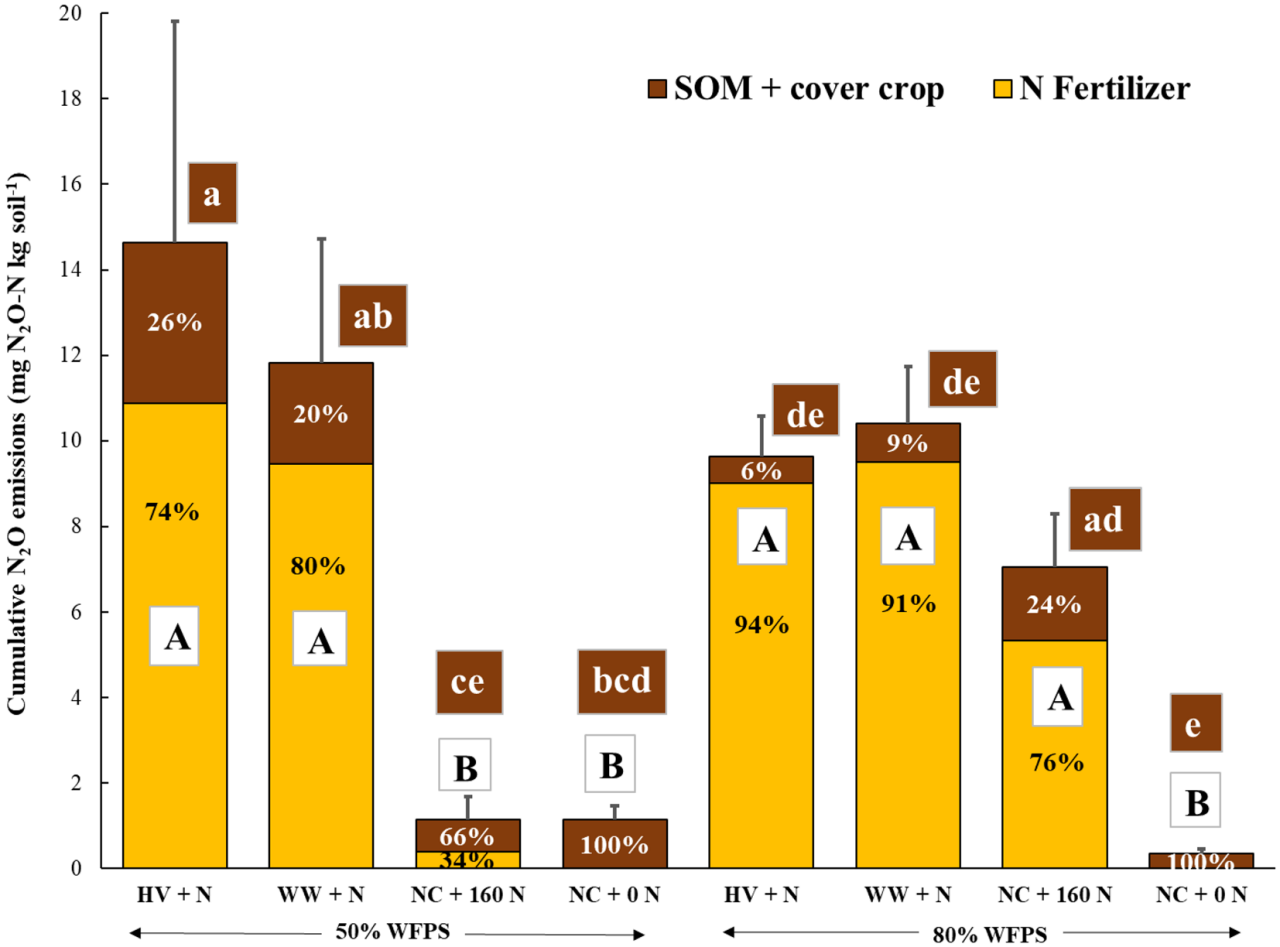
Cumulative N_2_O emissions over the experiment incubation period derived from the fertilizer (yellow) and soil organic matter and/or cover crops (brown) for four cover crops treatments at 50% and 80% WFPS. The error bars represent the mean standard errors for the total N_2_O emissions, and the percentage shown in the columns represents the proportion of cumulative total N_2_O emissions from each source. Uppercase letters indicate significant differences (*p* < 0.05) in cumulative N_2_O emissions and N_2_O emissions derived from N fertilizer among the treatments, while lowercase letters indicate differences in N_2_O emissions derived from SOM and/or cover crops among the treatments. HV, Hairy vetch; WW, Winter wheat; NC, No cover crop; NC + 0 N, Control.

Cumulative N_2_O and CO_2_ emissions reflected their daily emission patterns (Fig. 3 and Table 1). Irrespective of cover crop type, residue addition with N fertilizer under 50% WFPS produced statistically similar N_2_O emissions to 80% WFPS (Fig. 3, *p* > 0.05), known to facilitate large N_2_O emissions. However, 50% WFPS treatments with cover crop exhibited a greater variability of total N_2_O emissions compared to the corresponding treatments under 80% WFPS. Contrastingly, sole N fertilizer application increased N_2_O emissions only under 80% WFPS, the total emission being similar to the cover crop treatments (*p* > 0.05). Whereas N fertilization alone under 50% WFPS resulted in cumulative N_2_O emissions comparable to the control treatment (*p* > 0.05) and six times less than that in 80% WFPS (*p* < 0.05). Cover crop residue addition effect on CO_2_ emissions was more pronounced under 50% WFPS content and exhibited approximately twice as high cumulative CO_2_ emissions compared to the only N fertilizer and control treatments (*p* < 0.05) (Table 1). CO_2_ emissions were suppressed in all the treatments under 80% WFPS (*p* > 0.05).

**Table 1:** Cumulative CO_2_ emissions from all eight treatments after 21-day incubation.

Cover crop	WFPS (%)	Cumulative CO_2_ emissions (mg C–CO_2_ kg soil^−1^)
Hairy vetch + 160 N	50	382.8 (44.2)^ab^
Hairy vetch + 160 N	80	95.1 (13.1)^e^
Winter wheat + 160 N	50	472.4 (26.0)^a^
Winter wheat + 160 N	80	177.4 (17.7)^ce^
No cover crop + 160 N	50	207.9 (33.2)^bc^
No cover crop + 160 N	80	133.1 (24.9)^ce^
No cover crop + 0 N	50	199.8 (49.9)^cd^
No cover crop + 0 N	80	113.6 (45.1)^de^

*Values are means and standard errors in parenthesis. Lowercase letters indicate significant differences among the treatments (*p* < 0.05).

Fertilizer N was the main source of N_2_O across WFPS conditions, except for the only N fertilized treatment under 50% WFPS (Fig. 3). Under 80% WFPS, cover crop addition had little or no effect on N_2_O emissions derived from non-fertilizer sources such as soil organic matter (SOM) and/or cover crop. Cover crop treatments exhibited around 1.7 times higher fertilizer derived N_2_O emissions compared to the only N fertilized treatments under 80% WFPS. Cover crop addition had significantly (*p* < 0.05) greater N_2_O emission contribution from non-fertilizer (SOM and/or cover crop) sources under 50% than 80% WFPS. Under 50% WFPS, cover crop addition resulted in 23× and 3× higher N_2_O emissions derived from fertilizer and SOM and/or cover crop, respectively, than the N fertilized treatments. In presence of cover crop under 50% WFPS, N_2_O emissions derived from SOM and/or cover crop was 2× higher than that in the control treatment.

### Soil O_2_, C, and N availability

**Figure 4:**
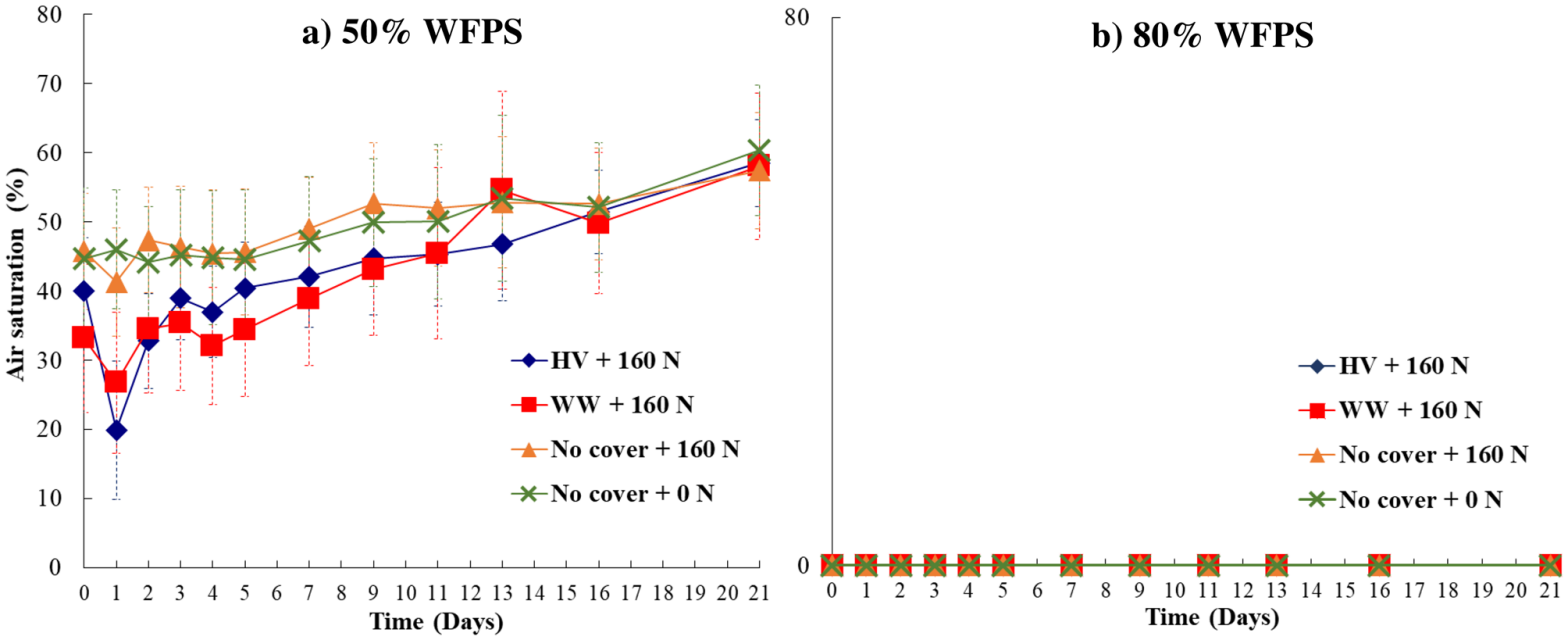
Average soil O_2_ content expressed as percentage of air saturation at 0 to 3 cm depth over the incubation period from four cover crops treatments at (**a**) 50% WFPS and (**b**) 80% WFPS. Bars represent the mean standard error. HV, Hairy vetch; WW, Winter wheat; No Cover, No cover crop; No cover + 0 N, Control.

**Figure 5:**
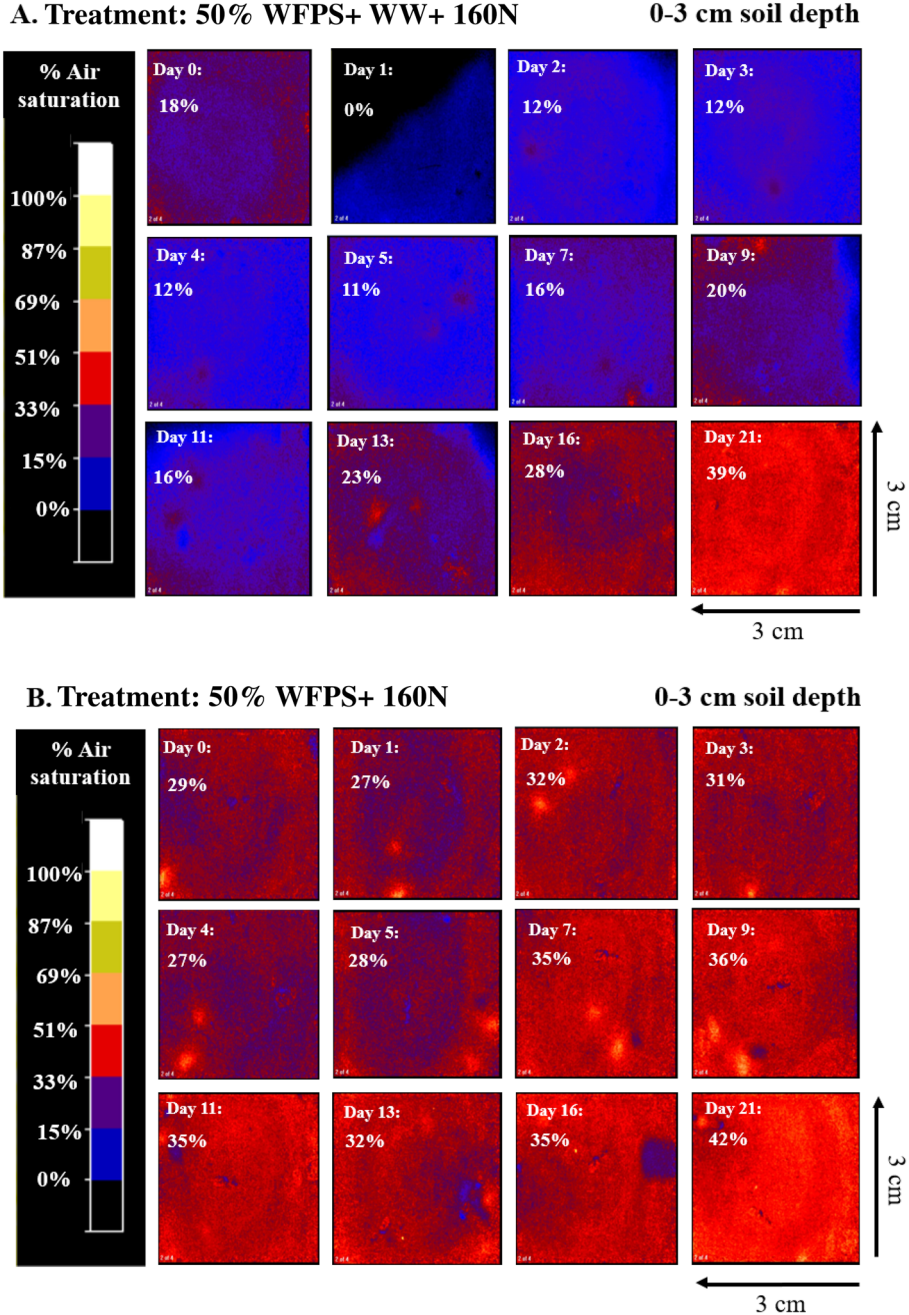
Selected images of O_2_ content expressed as percentage of air saturation in 0–3 cm soil depth over the incubation period from two treatments: (**A**) 50% WFPS with winter wheat + 160 N, (**B**) 50% WFPS with no cover crop + 160 N. Images (one of the four replicates) illustrate the maximum achieved O_2_ depletion effect resulting from cover crop addition.

Soil O_2_ concentration in the top 3-cm soil layer remained at anoxic levels during the whole incubation period for 80% WFPS treatments (Fig. 4b and Supplementary Fig. S2c). The 50% WFPS treatments with hairy vetch and winter wheat residues exhibited a sharp drop in O_2_ concentration by day 1 (mean air saturation of 20% and 27%, respectively), reaching anoxic soil O_2_ levels in some replicates (Fig. 5A and Supplementary Fig. S2a). Such a drop in soil O_2_ following cover crop residue incorporation under 50% WFPS also coincided with high CO_2_ emissions and concomitant onset of peak N_2_O emissions (Figs. 1a, 2a). Soil remained oxic throughout the incubation period under the N fertilized treatment without cover crop under 50% WFPS (Figs. 4a, 5B). Divergence in soil O_2_ between the 50% WFPS treatments with and without cover crops remained noticeable until around day 11 of the incubation experiment, and thereafter became comparable, with a much shorter duration for hairy vetch than winter wheat (Figs. 4a, 5, Supplementary Fig. S2a, b).

Prior to N fertilizer addition, all treatments had very low initial soil ${\text{NO}}_{3}^{ - }$ and ${\text{NH}}_{4}^{ + }$ concentrations (0.76 and 7.02 mg N kg^−1^, respectively). As expected, on day 2 after N addition, all N fertilized treatments showed significantly higher ${\text{NO}}_{3}^{ - }$ levels than the controls under each WFPS (*p* < 0.05), except for the 50% WFPS treatment with vetch (Table 2). In general, ${\text{NO}}_{3}^{ - }$ concentrations consistently decreased over time in all fertilized treatments under 80% WFPS, with only N fertilization maintained slightly higher ${\text{NO}}_{3}^{ - }$ levels for most of the sampling days. By day 7, 80% WFPS treatments with cover crop addition had ${\text{NO}}_{3}^{ - }$ concentrations comparable to the 80% WFPS control. In contrast, ${\text{NO}}_{3}^{ - }$ concentrations under 50% WFPS treatments with N addition either remained constant or slightly decreased over time and remained higher than that under 80% WFPS. Unlike ${\text{NO}}_{3}^{ - }$, soil ${\text{NH}}_{4}^{ + }$ concentrations decreased over time under 50% WFPS and the hairy vetch treatment had notably higher ${\text{NH}}_{4}^{ + }$ concentrations than the other treatments.

**Table 2:** Nitrate (${\text{NO}}_{3}^{ - }$) and ammonium (${\text{NH}}_{4}^{ + }$) concentration over the incubation period for four cover crop treatments at 50% and 80% WFPS.

Cover crop	WFPS (%)	NO_3_–N (mg N kg^−1^ soil)				
		Day 2	Day 7	Day 11	Day 16	Day 21
HV + 160 N	50	83 (17)^bd^	163 (26)^a^	133 (11)^a^	154 (30)^a^	147 (14)^a^
HV + 160 N	80	106 (34)^abc^	23 (7)^de^	5 (3)^c^	4 (2)^b^	1 (0)^d^
WW + 160 N	50	126 (18)^ab^	115 (13)^ac^	126 (19)^a^	114 (24)^a^	124 (9)^ab^
WW + 160 N	80	168 (24)^ab^	47 (19)^ce^	9 (1)^c^	8 (4)^b^	2 (0)^d^
NC + 160 N	50	179 (35)^a^	135 (27)^ab^	160 (9)^a^	124 (17)^a^	107 (5)^b^
NC + 160 N	80	166 (21)^ab^	80 (27)^bcd^	68 (22)^b^	32 (8)^b^	2 (1)^cd^
NC + 0 N	50	17 (1)^cd^	12 (1)^de^	10 (2)^c^	11 (2)^b^	26 (2)^c^
NC + 0 N	80	1 (0)^d^	2 (0)^e^	3 (1)^c^	2 (0)^b^	1 (0)^d^

Cover crop	WFPS (%)	NH_4_–N (mg N kg^−1^ soil)				
		Day 2	Day 7	Day 11	Day 16	Day 21
HV + 160 N	50	89 (3)^b^	31 (8)^b^	20 (3)^cd^	14 (5)^b^	6 (2)^c^
HV + 160 N	80	107 (6)^a^	86 (3)^a^	116 (5)^a^	93 (12)^a^	107 (9)^a^
WW + 160 N	50	14 (2)^e^	6 (1)^cd^	11 (2)^de^	5 (2)^b^	5 (1)^c^
WW + 160 N	80	36 (4)^c^	26 (6)^bd^	30 (2)^b^	11 (5)^b^	51 (4)^b^
NC + 160 N	50	18 (2)^de^	7 (1)^bd^	12 (1)^de^	3 (1)^b^	5 (1)^c^
NC + 160 N	80	31 (3)^cd^	28 (7)^bd^	26 (2)^bc^	12 (4)^b^	41 (2)^b^
NC + 0 N	50	13 (1)^e^	4 (0)^d^	5 (1)^e^	2 (1)^b^	4 (1)^c^
NC + 0 N	80	35 (0)^c^	29 (13)^bc^	22 (4)^bc^	14 (5)^b^	44 (3)^b^

HV, Hairy vetch; WW, Winter wheat; NC, No cover crop; NC + 0 N, Control.

*Values are means and standard errors in parenthesis. Lowercase letters indicate significant differences among the treatments (*p* < 0.05) within a column.

In general, soil POXC concentrations were higher in the 80% WFPS than the 50% WFPS treatments (mean value: 735 vs 677 mg kg soil^−1^), and the differences were only significant under the cover crop treatments on days 7, 11 and 21, and on day 2 in the hairy vetch treatment (Table 3). Cover crop residue addition had no significant effect on POXC concentrations when compared to the only N fertilized and control treatments under each WFPS condition.

**Table 3:** Soil permanganate oxidizable carbon (POXC) over the incubation period for four cover crop treatments at 50% and 80% WFPS.

Cover crop	WFPS (%)	POXC (mg C kg^−1^ soil)				
		Day 2	Day 7	Day 11	Day 16	Day 21
HV + 160 N	50	679 (24)^c^	686 (22)^c^	669 (11)^bc^	691 (15)^ab^	653 (20)^c^
HV + 160 N	80	795 (14)^a^	803 (13)^a^	744 (21)^a^	721 (9)^a^	784 (12)^a^
WW + 160 N	50	753 (15)^ac^	683 (19)^c^	648 (7)^c^	653 (20)^ab^	703 (31)^bc^
WW + 160 N	80	783 (15)^ab^	776 (6)^ab^	716 (31)^ab^	704 (21)^a^	791 (9)^a^
NC + 160 N	50	695 (9)^c^	684 (18)^c^	648 (11)^c^	660 (15)^ab^	712 (27)^ac^
NC + 160 N	80	743 (23)^ac^	702 (9)^bc^	694 (11)^ac^	653 (17)^ab^	685 (10)^bc^
NC + 0 N	50	706 (23)^bc^	665 (29)^c^	651 (13)^bc^	626 (8)^b^	671 (27)^c^
NC + 0 N	80	729 (29)^ac^	711 (17)^bc^	714 (19)^ab^	684 (37)^ab^	764 (20)^ab^

HV, Hairy vetch; WW, Winter wheat; NC, No cover crop; NC + 0 N, Control.

*Values are means and standard errors in parenthesis. Lowercase letters indicate significant differences among the treatments (*p* < 0.05) within a column.

### Drivers of N_2_O emissions

The Random Forest model explained daily N_2_O emission variations on the test data for 50% (R^2^ = 0.67, RMSE = 21.3) and 80% (R^2^ = 0.62, RMSE = 19.3) WFPS conditions (Table 4 and Supplementary Fig. S3). For 50% WFPS treatments, the model identified ${\text{NH}}_{4}^{ + }$ and O_2_ as the most influential variables impacting N_2_O emissions (Table 4). These variables collectively accounted for nearly 75% of the model’s performance, explaining 67% of the variance in N_2_O emissions on the test data set (Table 4). Conversely, at 80% WFPS, ${\text{NO}}_{3}^{ - }$ was the main driver of N_2_O emissions, and the model accounted for 62% of the N_2_O emissions variance on the test data set.

**Table 4:** Features importance to predict N_2_O emissions under 50% and 80% WFPS conditions as predicted by the Random Forest model.

Model variables	50% WFPS model	80% WFPS model
Ammonium (NH_4_^+^)	0.49	0.12
Nitrate (NO_3_^−^)	0.07	0.62
Oxygen (O_2_)	0.25	0.00
CO_2_	0.13	0.16
POXC	0.06	0.10
Model R^2^	0.67	0.62

Model R^2^ value indicates variability explained on the test data set.

*Values in the table rank the relevance of features in the model based on the Gini importance or mean decrease impurity.

## Discussion

Our findings revealed that even under suboptimal WFPS levels for denitrification (i.e., 50% WFPS in this study), N fertilized soils with cover crop residue addition exhibited N_2_O emissions of similar or higher magnitude than soils experiencing water-induced anoxia at 80% WFPS, widely reported to promote N_2_O emissions from denitrification^34,44,45^. This is in line with previous studies contradicting the conventional understanding that full pore saturation is a prerequisite for denitrification^37^. These findings carry notable implications for managing agricultural systems incorporating cover crops in the rotation to improve soil health through soil C sequestration. While elevated N_2_O emissions can offset the soil C sequestration benefits, our study underscores the importance of understanding the fundamental mechanisms of water-independent soil anoxia and controls of N_2_O emissions in response to cover crop management practices through the lens of high-resolution soil O_2_ measurements, a proximal driver of N_2_O emissions. This understanding is critical for accurate assessment of the net GHG mitigation potential of cover crops.

### Respiration induced anoxia during cover crop residue decomposition decouples WFPS control on N_2_O emissions

Mixing cover crop residues with N fertilizer created anoxia conducive for N_2_O production under 50% WFPS, which is otherwise well-aerated to limit large N_2_O emissions, especially from denitrification^21^. This led to high cumulative N_2_O emissions comparable with the respective treatments under 80% WFPS conditions (Fig. 3). On the other hand, the no cover crop treatments that received only N fertilizer under 50% WFPS produced N_2_O emissions as low as the control treatment and six times lower than that under primarily water-induced anoxia at 80% WFPS. These findings align with our first hypothesis which postulated that respiration-induced anoxia caused by decomposing cover crop residues can promote N_2_O emissions, even under sub-optimal WFPS conditions for denitrification. There are several lines of evidence that support this hypothesis. First, under 50% WFPS condition, cover crop residue addition exhibited a two-fold increase in cumulative CO_2_ production compared to the no cover crop and control treatments (Table 1). This indicates higher heterotrophic respiration from residue C mineralization in well-aerated conditions under moderate WFPS. Increased C availability, acting as energy source for the denitrifiers^24^, during cover crop residue decomposition was previously found to increase N_2_O emissions^28^. Such an effect was limited under the water-induced anoxia environment at 80% WFPS due to decreased overall residue decomposition. The divergence in temporal N_2_O emissions from the cover crop treatments under 50% and 80% WFPS conditions was evident up to day 5 of the incubation (Fig. 1a, b) indicating an accelerated phase of decomposition. Second, under 50% WFPS, peak N_2_O and CO_2_ emissions from the cover crop treatments coincided with depletion of soil O_2_ concentration two days following N fertilization (Figs. 1a, 2a, 4a). As previously reported in other studies^46,47^, the use of optode technology enables the visualization of highly-resolved spatial soil O_2_ dynamics following exogenous C incorporation. In the present study, the O_2_ images clearly demonstrated that within 24 h following N fertilization, the top 3 cm of soil experienced significant O_2_ depletion in treatments with cover crops and 50% WFPS (Figs. 4a, 5a), leading to the development of hypoxic or even anoxic conditions in certain replications. Such a mechanism to produce anoxia with a simultaneous increase in N_2_O will remain unexplained when WFPS alone is used as surrogate of soil O_2_ availability. Therefore, these findings further highlight the limitations of relying solely on WFPS in interpreting and predicting N_2_O fluxes, which can only account for biophysical mechanisms of anoxia resulting from O_2_ diffusion limitation in wet soils^48,49^, while neglecting anoxia caused by microbial O_2_ consumption during residue decomposition^20^.

Cover crop incorporation led to high N_2_O emissions in 50% WFPS soils, which can pose a greater environmental risk compared to the same emissions in soils under water-induced anoxia. This is due to the higher relative gas diffusivity of N_2_O in soil versus air under 50% WFPS, with air-filled macropores, compared to 80% WFPS. The higher diffusivity would facilitate rapid escape of N_2_O from the soil^49,50^, reducing the chances of biological N_2_O reduction to N_2_. Under 80% WFPS, a greater potential exists for further reduction of N_2_O to N_2_ before the gas escapes from the soil into the atmosphere^51^. This scenario is particularly plausible in our study due to the depletion of substrates with a higher redox reaction energy yield, such as soil ${\text{NO}}_{3}^{ - }$ supply under 80% WFPS (Table 2). In response, it is conceivable that microbes resort to using a redox couple with a lower energy yield, forcing greater N_2_O reduction to N_2_^24^.

### Cover crop residue influenced nitrogen fertilizer derived N_2_O losses under different WFPS

The fraction of fertilizer derived N_2_O was greater under the co-presence of cover crops and N fertilizer compared to N fertilization with no cover crops, irrespective of WFPS status. However, a greater fraction of N_2_O emissions was derived from SOM and/or cover crop sources under 50% than 80% WFPS (Fig. 3). This may be explained by the additional N mineralized from the cover crop residues and the native SOM pool under 50% WFPS contributing to soil ${\text{NH}}_{4}^{ + }$ and ${\text{NO}}_{3}^{ - }$ pools that are substrates for N_2_O production^9^. Depletion of the ${\text{NH}}_{4}^{ + }$ pool over time and relatively high ${\text{NO}}_{3}^{ - }$ levels sustained throughout the incubation period indicates that nitrification of ${\text{NH}}_{4}^{ + }$ released from mineralization of cover crop residues and SOM was continued under 50% WFPS (Table 2). The mineral N trend was in sharp contrast with the cover crop treatments under 80% WFPS where once the available ${\text{NO}}_{3}^{ - }$ from N fertilizer was exhausted, the system became ${\text{NO}}_{3}^{ - }$ limited due to inhibited nitrification of SOM and/or cover crops under wetter soils^34^. This was also reflected by high ${\text{NH}}_{4}^{ + }$ availability sustained throughout the experiment. Therefore, the additional N sources, other than N fertilizer, under 50% WFPS perhaps resulted in three to six times higher N_2_O contribution from the SOM and/or cover crop residues than that under 80% WFPS.

### Differential drivers and processes of N_2_O emissions in response to cover crops and WFPS

The patterns of daily N_2_O emissions differed among treatments with cover crop inclusion at 50% and 80% WFPS content, associated with distinct drivers of N_2_O emissions in each case (Fig. 1a, b). Under 80% WFPS content, peak N_2_O emissions for treatments with cover crops were exhibited at the initial stages of the incubation experiment until ${\text{NO}}_{{3{ }}}^{ - }$ became a limiting factor (Table 2). The Random Forest model indicated a strong association between ${\text{NO}}_{3}^{ - }$ levels and N_2_O emissions under 80% WFPS, where ${\text{NO}}_{3}^{ - }$ was the most important feature in a model that accounted for 62% of the variability in N_2_O emissions (Table 4 and Supplementary Fig. S4). Under water induced anaerobic conditions at 80% WFPS, the N_2_O production was likely governed by denitrification primarily dependent on ${\text{NO}}_{3}^{ - }$ from the fertilizer, while nitrification was restricted^52^. This was indicated by the small change in soil ${\text{NH}}_{4}^{ + }$ over the incubation period, particularly in the treatments with hairy vetch (Table 2). Consequently, approximately 90% of the N_2_O emissions were derived from NO_3_-N fertilizer from day 0 to day 5 of the incubation experiment for treatments with cover crops (Fig. 1d). The restriction in nitrification resulted in consistently low N_2_O emissions once fertilizer ${\text{NO}}_{3}^{ - }$ levels were exhausted (Fig. 1b, Table 2). The low importance assigned to soil O_2_ concentration in the Random Forest model to explain the variability in N_2_O emissions under 80% WFPS highlights that NO_3_-N availability becomes the main driver under reduced gas diffusivity.

Conversely, N_2_O emissions under 50% WFPS were heavily related to ${\text{NH}}_{4}^{ + }$, soil O_2_, and CO_2_ emissions as these parameters were the main features in a model that explained 67% of the variability in N_2_O emissions (Table 4 and Supplementary Fig. S5). Peak N_2_O emissions for treatments with cover crop coincided with elevated soil CO_2_ emissions and depletion of soil O_2_ contents two days following N fertilization (Figs. 2a, 4a). During the peak emissions, N_2_O fluxes were primarily derived from readily available NO_3_-N from N fertilizer (75–91%, Fig. 1c), suggesting that even under 50% WFPS, a large proportion of the total N_2_O emissions in the early stages of the incubation experiment were from denitrification^53,54^. This finding is consistent with previous existing studies, which identified a decoupling of the conventional relationship between WFPS and N_2_O production pathways^36,37^. Similar to these studies, our findings contrast with the conceptual model of sources of N_2_O presented by Davidson et al.^34^, which proposes nitrification as the main source of N_2_O under 60% WFPS . However, denitrification was not the sole process contributing to N_2_O emissions under 50% WFPS. A declining soil ${\text{NH}}_{4}^{ + }$ pool with a sustained supply of ${\text{NO}}_{3}^{ - }$ over the incubation period (Table 2), along with a reduction in the fraction of N_2_O derived from fertilizer (Fig. 1c) but with a sizable amount of total N_2_O production after the initial peak emission phase, suggests that nitrification^52^ or even nitrifier denitrification^55^ might have also contributed to N_2_O fluxes, and accounted for the importance of the ${\text{NH}}_{4}^{ + }$ feature in the Random Forest model.

While ${\text{NO}}_{3}^{ - }$ drove N_2_O emissions in all treatments under 80% WFPS conditions, the N_2_O daily emission pattern and magnitude differ between treatments with and without cover crop residues addition (Fig. 1b). In residue amended soils, peak N_2_O emissions were approximately three-fold higher than those without residue, evidencing that the exogenous addition of labile C triggered N_2_O emissions. As previously documented, these results confirm that C limitation to microbes is a crucial driver for heterotrophic denitrification^56,57^, particularly under water-induced anoxia and high soil ${\text{NO}}_{3}^{ - }$ levels, and that cover crop residues can overcome such limitation to add to the risks of enhanced N_2_O emissions^12,58^.

The occurrence of respiration-induced anoxia under 50% WFPS is expected to be closely linked with organic matter mineralization^59^. Peak N_2_O and CO_2_ emissions were closely associated when cover crop residues were added under 50% WFPS (Figs. 1a, 2a). A recent study by Ye et al.^60^, showed that straw residue incorporation affected soil N_2_O and CO_2_ emissions by altering the dissolved organic carbon and O_2_ content of the soil. A greater association of CO_2_ than POXC with N_2_O emissions (Table 4), especially under 50% WFPS, highlights that microbial respiration is closely linked to soil CO_2_ emissions, while POXC represents net C availability.

### Cover crop type did not significantly influence N_2_O emissions under both 50 and 80% WFPS

Irrespective of the WFPS content, there was no significant difference in cumulative N_2_O production between legume and non-legume cover crops (hairy vetch vs winter wheat, Fig. 3). This finding does not support our second hypothesis, which proposed that co-locating mineral N fertilizer and high-quality vetch cover crop residues would deplete soil O_2_ at a faster rate compared to grass wheat cover crop residues, resulting in greater N_2_O emissions. This finding contrasts with several studies that have demonstrated higher N_2_O emissions via denitrification from legume residues compared to grass residues, across different WFPS content^10,54,61^. This discrepancy between studies can be attributed to the following reasons: First, due to the mixing of residues with high level of N fertilizer, ${\text{NO}}_{3}^{ - }$ levels were never limiting throughout the entire incubation period under 50% WFPS (Table 2). Thus, the ${\text{NH}}_{4}^{ + }$ supply from vetch residue decomposition and the subsequent N_2_O emissions derived from nitrification were not sufficient to significantly differentiate cumulative N_2_O emissions between treatments with hairy vetch and winter wheat. Second, soil O_2_ levels during the incubation period exhibited similar patterns between the different cover crop types under 50% WFPS content (Fig. 4a), suggesting that the easily decomposable C fraction did not differ significantly between treatments. Third, winter wheat C:N ratio (24:1) was not high enough to induce net N immobilization and reduce soil N_2_O emissions^20,62,63^.

## Conclusions

Our study illustrates the biogeochemical mechanism of anoxia formation by accelerated microbial respiration following cover crop residue and N fertilizer addition to influence N_2_O emissions. This was achieved by using a novel planar optode sensing technology, enabling high-resolution measurement of soil profile O_2_ dynamics in response to residue addition. Under suboptimal WFPS levels for denitrification, N_2_O emissions can be triggered by cover crop residues to a similar or even higher level than in soils experiencing water-induced anoxia that typically promotes large N_2_O emissions from denitrification. Under water induced anaerobic conditions (80% WFPS), cover crops controlled N_2_O emissions via altering labile C availability and had little effect on mineral N availability. Whereas under relatively aerobic conditions (50% WFPS), cover crop residue decomposition consumed soil O_2_ to promote anoxia that led to increased N_2_O emissions. This scenario poses a greater environmental risk compared to soils under water-induced anoxia as it enables the rapid escape of N_2_O from soil due to higher diffusivity and reduces the likelihood of biological N_2_O reduction to N_2_. These findings hold crucial implications for managing agricultural systems using cover crops. The respiration-induced anoxia mechanism observed in this study along with cover crop’s role in altering coupled soil C, N, and water cycling will drive net soil N_2_O emissions which should be accounted for within the broader context of assessing cover crop impacts on soil health. Elevated N_2_O emissions can offset the benefits of soil C sequestration, often intended when using cover crops. This further highlights the importance of accurately assessing the C footprint of cover crops by quantifying their impacts on N_2_O emissions.

The decoupling of WFPS controls on soil O_2_ can be prominent as decomposition rate increases. This poses a formidable challenge in accurately predicting N_2_O emissions, particularly in the context of growing adoption of cover cropping for soil health. Our study suggests that the occurrence of respiration-induced anoxia during cover crop residue decomposition in fertilized soils can disrupt the traditional WFPS controls on N_2_O emissions. Relying solely on WFPS, an imperfect proxy for diffusion-induced O_2_ limitation, may lead to the underestimation and inaccurate prediction of potential risks associated with N_2_O emissions, especially in cover crop based fertilized agricultural systems. Therefore, models should incorporate a more comprehensive understanding of these dynamics to enhance predictive accuracy and better capture the complexities of N_2_O emissions in such agroecosystems.

## Materials and methods

### Experimental site and soil sampling

Surface soil samples (0–10 cm depth) were collected from a no-till corn (*Zea mays)* field at the University of Tennessee’s West Tennessee Research and Education Center in Jackson, Tennessee (35° 37′ 22″ N, 88° 50′ 47″ W; elevation 125 m), United States, in August 2022. A total of 50 kg of dry soil were randomly collected from four replicated plots maintained without N fertilization for two years to achieve a low background soil N level. The study site soil is classified as a Lexington silt loam (fine-silty, mixed, thermic Ultic Hapludalfs), organic matter was 15.5 g kg^−1^, total N was 0.85 g kg^−1^, and pH (H_2_O) was 6.3. The moist soil was thoroughly mixed, air dried, sieved (< 6 mm), and stored at 21 °C until the experiment started. At the start of the incubation experiment, the soil contained 0.6 mg kg^−1^ ${\text{NO}}_{3}^{ - }$ and 7.1 mg kg^−1^ ammonium-N $\left( {{\text{NH}}_{4}^{ + } } \right)$.

### Cover crop biomass sampling

Two cover crops were included in this study, (1) hairy vetch (*Vicia villosa*), which is a legume with a low C/N ratio (10:1), and (2) winter wheat (*Triticum aestivum L.*), which is a cereal with a relatively higher C/N ratio (24:1). Above ground cover crop biomass was sampled at approximately 2 cm above the soil surface in April 2022, just before cover crop termination. Oven dried (60 °C for 48 h) samples were cut into 5 mm pieces and stored until the start of the incubation experiment. Total C and N concentrations of ground residue subsamples were determined using an Elementar vario Max cube CN analyzer (Elementar, Hanau, Germany).

### Incubation experimental set up

A three-week long incubation experiment was established in a randomized complete block design with three factors: two levels of N addition (control 0 N and equivalent rate of 160 kg N ha^−1^ as K^15^NO_3_ at 10 atom % excess), three levels of cover crop residue addition (hairy vetch, winter wheat, and no cover crop) and two levels of WFPS (50% and 80%). The experimental design resulted in eight treatments as follows: T1: 50% WFPS + hairy vetch + 160 kg N ha^−1^, T2: 50% WFPS + wheat + 160 kg N ha^−1^, T3: 50% WFPS + no cover crop + 160 kg N ha^−1^, T4: 50% WFPS (0 N and no cover crop addition as Control), T5: 80% WFPS + hairy vetch + 160 kg N ha^−1^, T6: 80% WFPS + wheat + 160 kg N ha^−1^, T7: 80% WFPS + no cover crop + 160 kg N ha^−1^, and T8: 80% WFPS (0 N and no cover crop addition as Control). Cover crop residues were added at an equivalent rate of 3 Mg dry matter (DM) ha^−1^, a typical biomass production for spring cover crops in Tennessee under desirable weather conditions. Four replicates were prepared for each of the 8 treatments and four additional sets of samples were included for five-time points destructive soil samplings, with a total of 160 (32 for gas sampling + 128 for destructive soil sampling) experimental units.

Soil cores (5 cm w × 5 cm l × 10 cm h) were packed at a bulk density of 1.2 g cm^−3^ in 15 cm long rectangular transparent acrylic liners. The soil and cover crops residues were weighed (300 g dry soil and 0.750 g DM residue equivalent to 3 Mg DM ha^−1^ for treatments receiving cover crops), mixed, and added to each acrylic liner. Experimental units were pre-incubated in the dark for 72 h with a soil water content amended to achieve the targeted 50% and 80% WFPS, saving 10 mL of water to dissolve the N fertilizer. Water was added from the bottom end of the cores, which were sealed with parafilm with small holes to prevent soil leakage but allows wetting through capillary rise. The top end of plastic liners was covered with perforated parafilm to minimize evaporation, which was removed 1 h before gas sampling.

At the start of incubation, 0.289 g K^15^NO_3_ (10 atom % excess ^15^N), equivalent to the recommended 160 kg N ha^−1^, was dissolved in 10 mL de-ionized water and applied to the fertilized treatments with a syringe from the top (133 mg N kg^−1^ soil was added as KNO_3_). We used K^15^NO_3_ as fertilizer source to facilitate testing our hypothesis that anoxia from residue decomposition would promote N_2_O production from denitrification (i.e., reduction of ${\text{NO}}_{3}^{ - }$) independent of WFPS conditions. All experimental units were incubated in the dark at 21 °C until gas sampling for 21 consecutive days and kept at a constant %WFPS level for the duration of the incubation experiment by adding water based on daily weight losses of the cores.

### Gas sampling, analysis, and N_2_O source calculations

Gas samples were taken on days 0 (12 h after N fertilizer addition), 1, 2, 3, 4, 5, 7, 9, 11, 13, 16, and 21. Each core was transferred to a 2 L jar and closed with a lid that contains a septum for gas sampling. Headspace samples (120-mL) were withdrawn from the jars at 60 and 120 min after closure. Three additional samples of lab air were taken each sampling day. The 60 mL syringe was plunged 3 times to mix the gas in the 2L chamber before final collection. The collected gas samples were injected into pre-evacuated 100 mL crimp top clear serum vials and analyzed for CO_2_ and N_2_O on a Delta + XL mass spectrometer (Thermo Finnigan) coupled with a Precon and Gasbench II (ThermoScientific). Overpressure in the vials allowed for sequential sampling of the gas sample for the two gases. CO_2_ was measured and then the overpressure in the jar was vented. The entire volume of the vial gas was then transferred to a liquid N cooled trap for N_2_O measurement.

The fluxes of CO_2_ and N_2_O were calculated from the increasing gas concentrations during the 120-min headspace closure. The cumulative gas emissions during the 21-day experiment were calculated by linear interpolation of daily emissions.

The ^15^N abundance in N_2_O was determined using the same headspace samples. The relative contributions of N_2_O by fertilizer-N (f_N2O_fertilizer_) and other-N sources (SOM + cover crop residues, f_N2O_Other_) were determined with a mixing model as follows:

1 $${\text{f}}_{{{\text{N}}_{2} {\text{O}}\_{\text{fertilizer}}}} + {\text{f}}_{{{\text{N}}_{2} {\text{O}}\_{\text{Other}}}} = 1$$

2 $$\updelta ^{15} {\text{N}}_{{{\text{Treatment}}}} =\updelta ^{15} {\text{N}}_{{{\text{fertilizer}}}} \times {\text{f}}_{{{\text{N}}_{2} {\text{O}}\_{\text{fertilizer}}}} +\updelta ^{15} {\text{N}}_{{{\text{Other}}}} \times {\text{f}}_{{{\text{N}}_{2} {\text{O}}\_{\text{Other}}}}$$

where δ^15^N_Treatment_ is the measured δ^15^N of the total N_2_O, δ^15^N_fertilizer_ is the δ^15^N of KNO_3_ (10 atom % excess ^15^N), and δ^15^N_Other_ is the δ^15^N in the N_2_O produced by the SOM and/or cover crop residues. Since the natural enrichment in ^15^N between soil and cover crop residues is small compared to the ^15^N enrichment in the labelled N fertilizer, the isotopic composition of the N_2_O produced by the other sources was assumed to be the value measured in the control treatments (T4 and T8).

### Soil O_2_ measurement

Spatially resolved soil O_2_ dynamics, measured as % air saturation, was monitored using planar optode technology of VisiSensTM A1 system (PreSens GmbH, Germany) with a portable detector unit DU01 containing a Universal Serial Bus (USB) microscope^64–66^. Each incubation core for gas sampling had an O_2_ sensor foil (SF-RPSu4) attached to the inner side of the acrylic liner wall throughout the length of the 10-cm soil column that allowed the high-resolution 2-D imaging of soil O_2_ saturation. Briefly, the O_2_ sensor foil contains fluorescent dyes sensitive to soil O_2_ concentration that, when exposed to the LED light from the detector unit, emit fluorescence of specific wavelengths that are captured by the microscope, which translates the data into color images. The images were taken immediately after gas sampling and under dark conditions. Prior to starting the measurements, calibration was performed with identical ambient and temperature conditions as experimental readings. Calibration was performed using a two-point calibration method as recommended by the manufacture’s instruction manual, where a solution of oxygen-free water (0% air saturation) was used as first calibration point and ambient air (100% air saturation) was used as second calibration point. Three images were captured in each acrylic liner by placing the detector in the sensor foil at 0–3, 3–6, and 6–9 cm depths to ensure the measurement of real-time high resolution spatial distribution of O_2_ along the soil profile. Image processing was performed using ViSiens Imaging System Software (version VA1.12). A graphical description of the measurement setup is provided in Supplementary Fig S1. For a more detailed description, refer to Keiluweit et al.^59^.

Based on O_2_ content, soils O_2_ can be categorized into three groups as in Wang et al.^46^: oxic (> 2.00 mg L^−1^, equivalent air saturation > 22.5%), hypoxic (0.14 to 2.00 mg L^−1^, equivalent air saturation 1.6 to 22.5%) and anoxic (< 0.14 mg L^−1^, equivalent air saturation < 1.6%) conditions.

### Soil analysis

On days 2, 7, 11, 16, and 21, soil cores were destructively sampled and analyzed for soil inorganic N (${\text{NH}}_{4}^{ + }$ and ${\text{NO}}_{3}^{ - }$) content by 2 M KCl extraction^67,68^ and permanganate oxidizable C (POXC)^69^.

### Statistical analysis

Statistical analyses were conducted using R statistical software version 4.3.0 (2023)^70^ with a significance level set at *p* < 0.05. The ANOVA model included treatments (a combination of cover crop, N fertilization, and WFPS content) as fixed effects, and block as a random effect. Following significant ANOVA results, Tukey´s honest significant difference (HSD) test was performed to compare treatments for cumulative CO_2_ and N_2_O emissions, as well as soil O_2_, ${\text{NH}}_{4}^{ + }$, ${\text{NO}}_{3}^{ - }$, and POXC concentrations. Data were transformed using Box-Cox, logistic, or square root transformations, as appropriate, to meet the assumptions of normality and homoscedasticity. Throughout the paper, the error terms reported are the mean standard errors.

Random Forest regression model^71^ was used to understand the differential controls of N_2_O emissions under 50% and 80% WFPS. The dataset was separately analyzed for 50% and 80% WFPS treatments (n = 352 for each WFPS). The input variables included CO_2_, O_2_, ${\text{NH}}_{4}^{ + }$, ${\text{NO}}_{3}^{ - }$, and POXC, with the aim of identifying the main drivers of N_2_O emissions and evaluating model performance. Briefly, each dataset was split into training (80% data, n = 281) and testing (20% data, n = 71) data, with the training set used to train the Random Forest model using the model parameters as follows: random_state = 42 and n_estimators = 100. The importance of predictor variables was assessed for 50% and 80% WFPS conditions, and model performance was evaluated on the test data set using RMSE and R^2^ model metrics. The Random Forest model was fitted using “RandomForestRegressor” function from the “sklearn.ensemble” modules in Python (Python 3.10).

### Supplementary Information

Supplementary Information.

## Data Availability

The datasets used and analyzed during the current study will be made available by the authors upon reasonable request to the corresponding author (Debasish Saha, dsaha3@utk.edu).
