# Supplementary material for: Cover crop residue decomposition triggered soil oxygen depletion and promoted nitrous oxide emissions
Source: Sci Rep. 2024 Apr 10;14:8437. doi: 10.1038/s41598-024-58942-7 (PMC11006885; doi:10.1038/s41598-024-58942-7)
Supplement: Supplementary file 1 — Supplementary Information. [file 41598_2024_58942_MOESM1_ESM.docx]

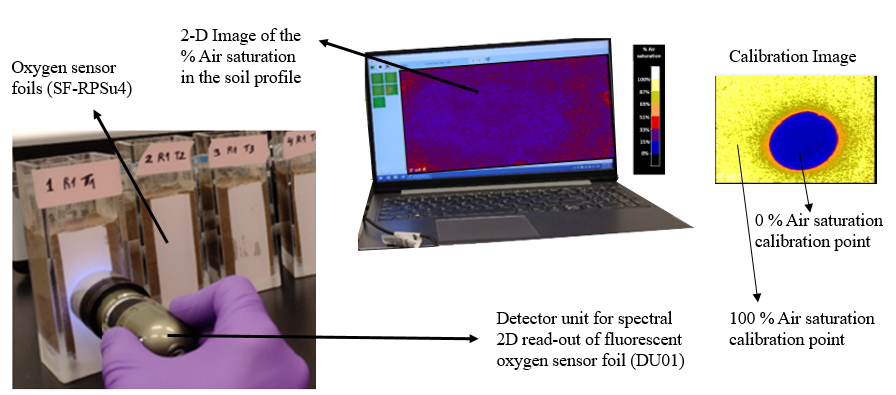


**Supplementary Figure S1.** Illustration of the experimental set-up (not to scale) and calibration image.


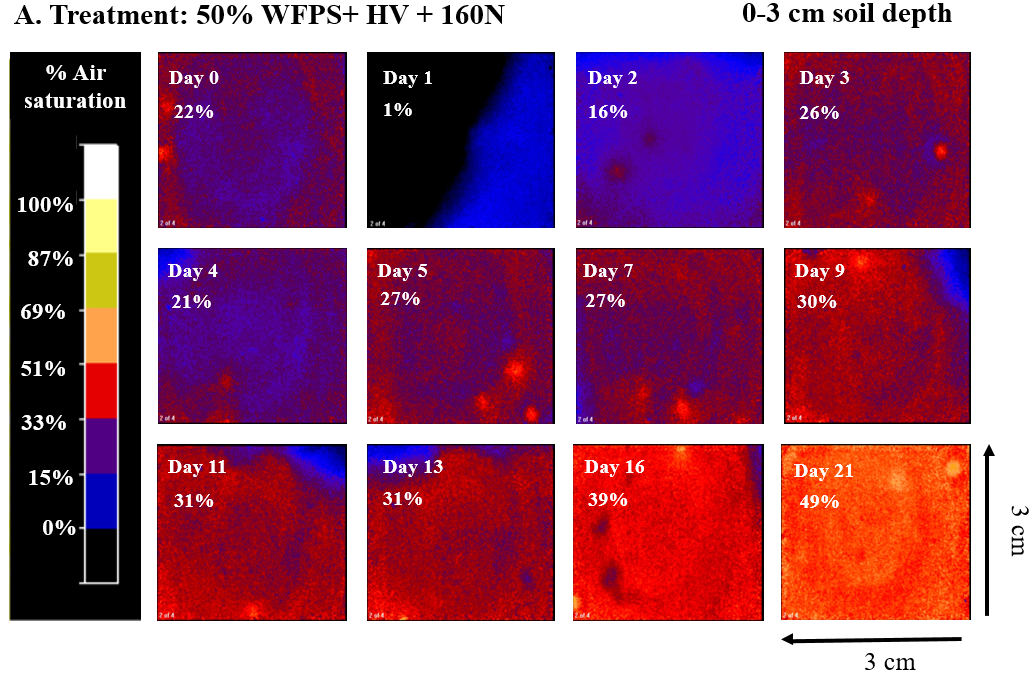


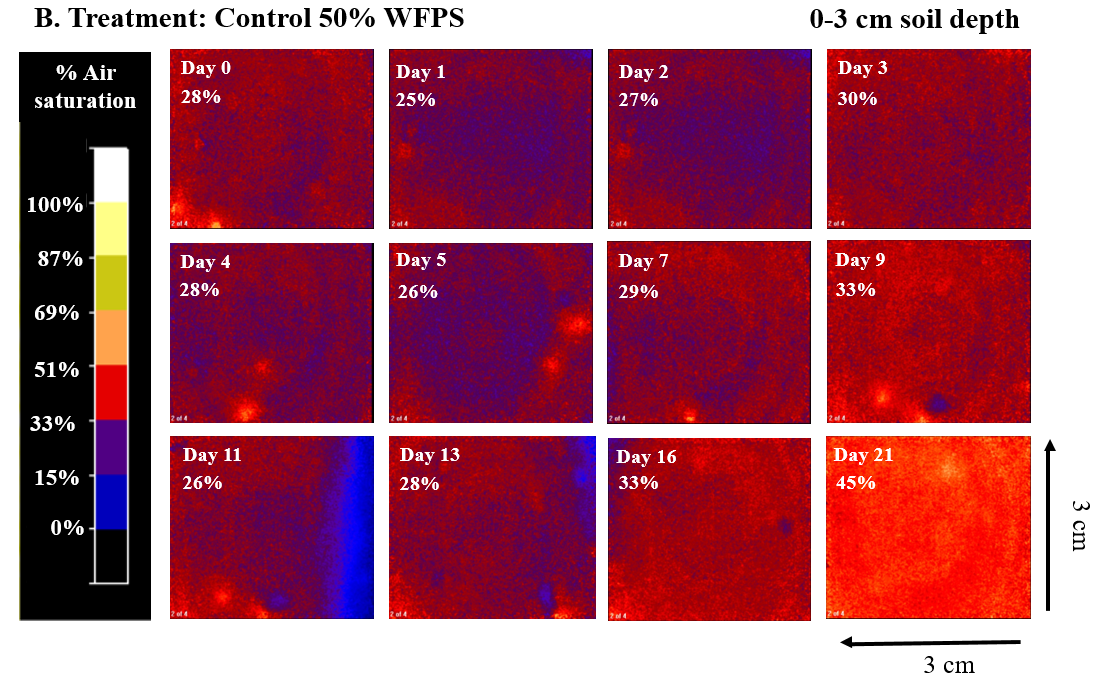


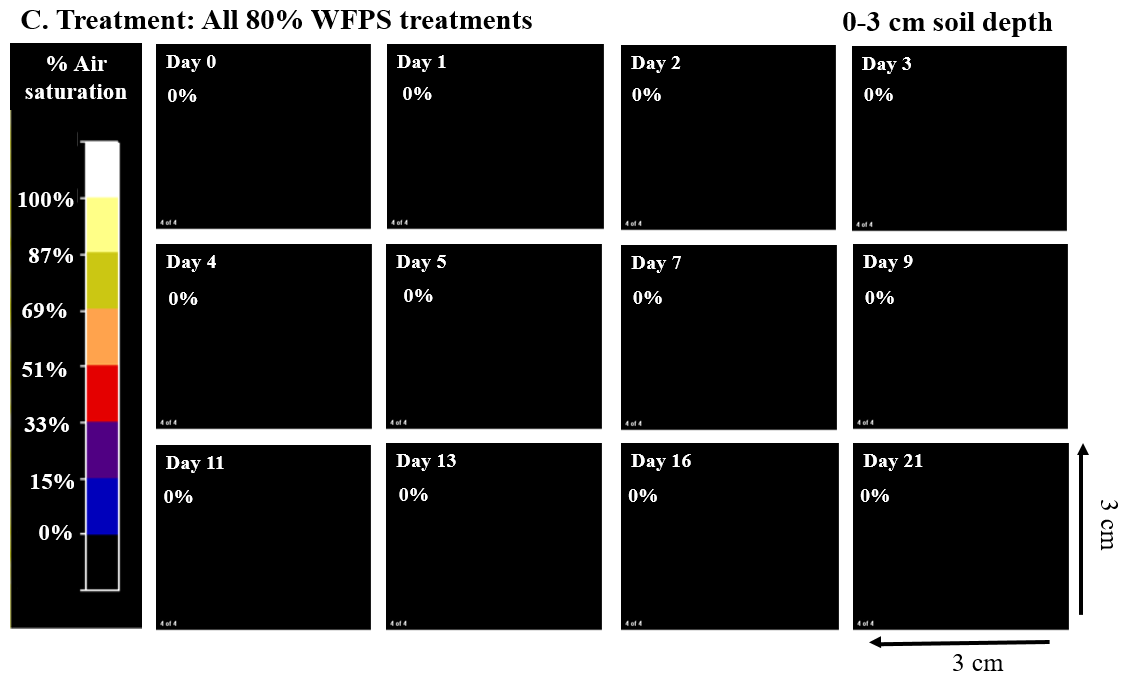


**Supplementary Figure S2.** Selected images of O_2_ content expressed as percentage of air saturation in 0 to 3 cm soil depth over the incubation period from different treatments: A) 50% WFPS with hairy vetch, B) 50% WFPS control, and C) all 80% WFPS treatments. Images (one of the four replicates) are an example of the effect of the different treatments.


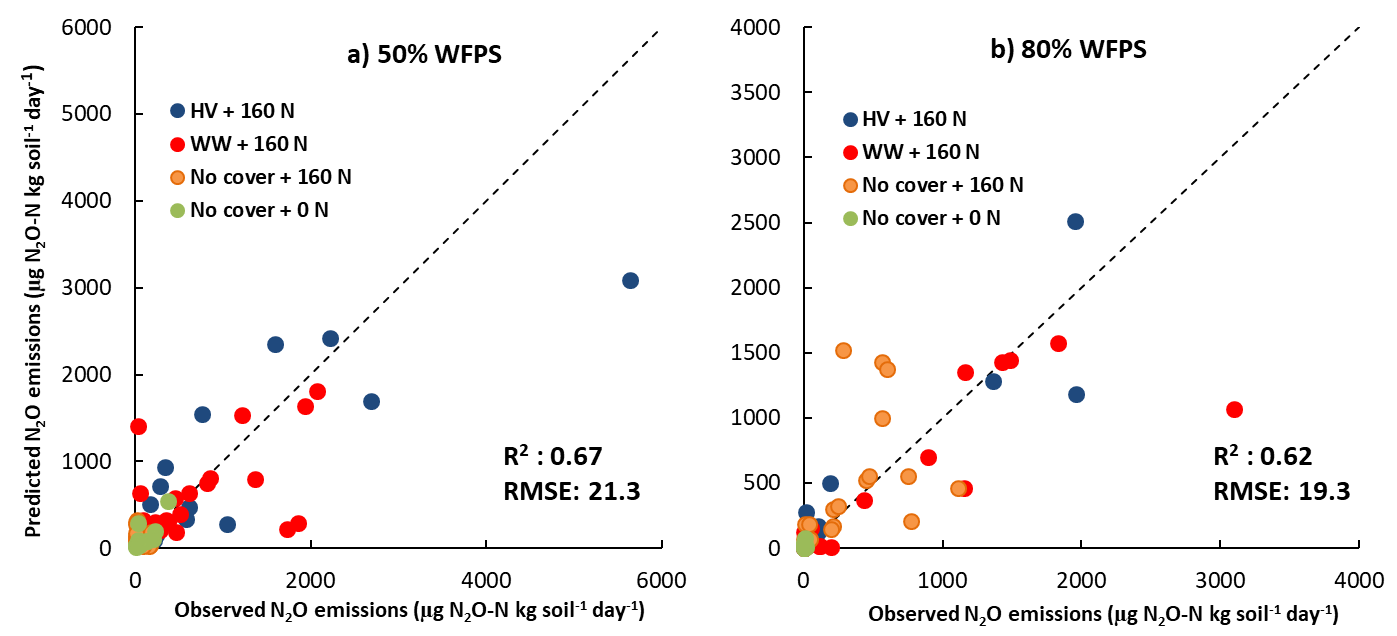


**Supplementary Figure S3.** Predicted vs observed N_2_O emissions from the Random Forest N_2_O flux model on the test data set: a) 50% WFPS and b) 80% WFPS. HV: Hairy vetch, WW: Winter wheat, No cover: No cover crop, No cover + 0 N: Control


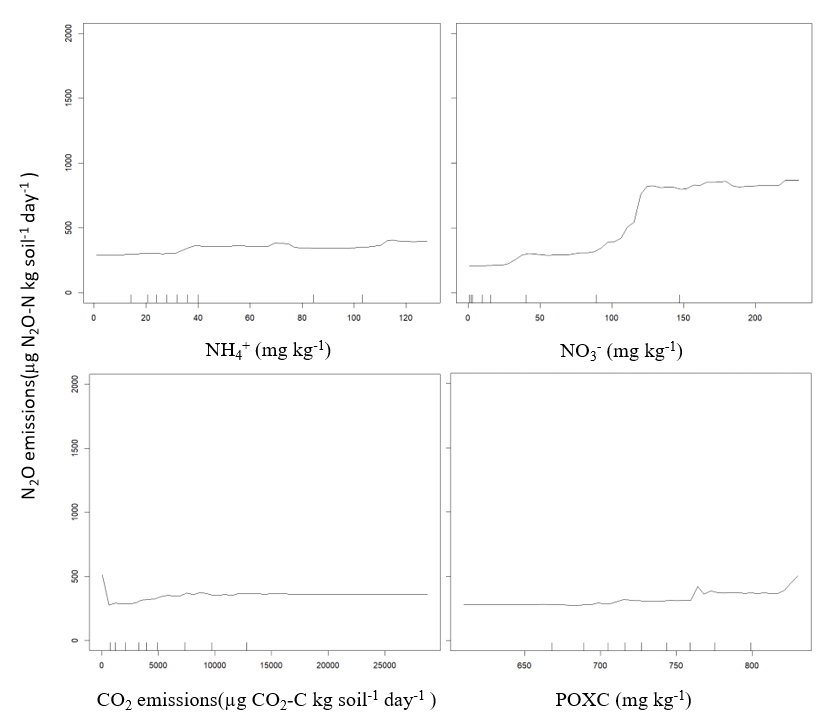


**Supplementary Figure S4.** One dimensional partial dependence of predictor variables on N_2_O emissions as predicted by Random Forest model under 80% WFPS.

**
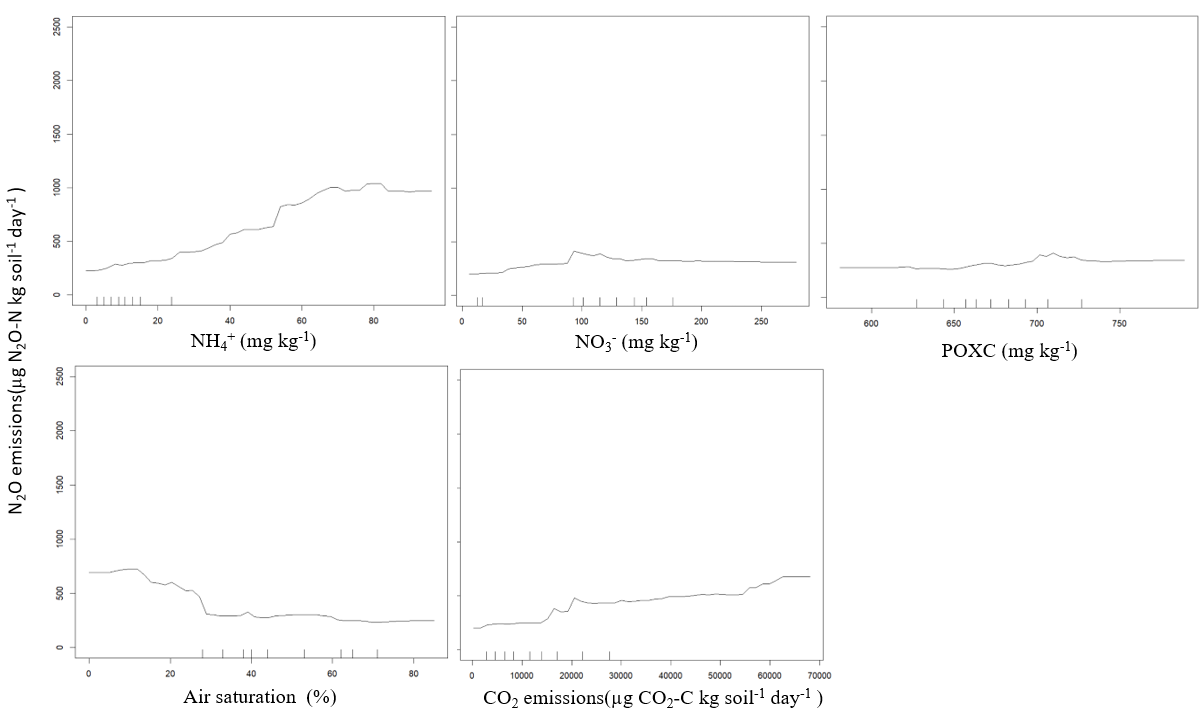
**

**Supplementary Figure S5.** One dimensional partial dependence of predictor variables on N_2_O emissions as predicted by Random Forest model under 50% WFPS.
